# Social Anxiety and Orthognathic Surgery Effect on Oral Health-Related Quality of Life

**DOI:** 10.7759/cureus.45434

**Published:** 2023-09-18

**Authors:** Mohamed Bamashmous, Majed Zahran, Anaan Bushnag, Mohammed A Sindi, Heba Ashi, Dania Sabbahi, Shoroog Agou, Fahad Alsulaimani

**Affiliations:** 1 Dental Public Health, King Abdulaziz University, Jeddah, SAU; 2 Orthodontic and Dentofacial Orthopedics, Boston University, Boston, USA; 3 Prosthodontics, Imam Abdulrahman Bin Faisal University, Dammam, SAU; 4 General Dentistry, Ministry of Health, Turaif, SAU; 5 General Dentistry, King Abdulaziz University, Jeddah, SAU; 6 Orthodontics, King Abdulaziz University, Jeddah, SAU

**Keywords:** social anxiety, questionnaire, quality of life, orthognathic surgery, ohrqol

## Abstract

Objectives: This study aimed to investigate the impact of patients' social anxiety on oral health-related quality of life (OHRQoL) in the context of orthognathic surgery.

Methods: The study involved a cohort of 70 patients who were tasked with completing a comprehensive questionnaire aimed at assessing various facets of their OHRQoL at distinct stages of treatment: pre-surgery, within one month post surgery, and more than one month post surgery. Statistical analyses were conducted in the form of t-test.

Results: Out of the 70 participants, 27 were male (38.57%) and 43 were female (61.43%). The sample distribution comprised 30 (43%) subjects in the pre-surgical stage, two (3%) at one month post surgery, and 38 (54%) more than one month post surgery. In relation to the t-test results, we found variations in the significance of the results for each question, with multiple results showing patients who reported experiencing discomfort significantly outnumbering those who did not (p < 0.05).

Conclusion: Social anxiety and orthognathic surgery both demonstrate significant influences on OHRQoL. Subsequent research should delve into specific areas where patients experience the greatest impact.

## Introduction

Oral health-related quality of life (OHRQoL) refers to the absence of adverse consequences stemming from oral conditions on an individual's social life and their positive perception of dentofacial self-assurance. OHRQoL encompasses various dimensions, including dental esthetics, facial appearance, articulation clarity, masticatory competence, the ability to express emotions through laughter, respiratory comfort, painless functioning, as well as social and physical well-being [1,2]. This multifaceted assessment serves the purpose of evaluating the impact of chronic oral diseases and the efficacy of treatment modalities. Additionally, OHRQoL augments clinical data by offering a comprehensive evaluation [3-5].

Orthognathic surgery profoundly influences an individual's quality of life (QoL) by reinstating normative oral function and enhancing facial and oral aesthetics [6,7]. Nevertheless, patients often grapple with post-operative adjustments, occasionally encountering novel challenges that surpass those experienced prior to the surgical intervention. Potential complications, such as facial edema and altered dietary patterns, can trigger psychological repercussions, potentially culminating in anxiety or depression. Consequently, the enhancement of QoL and the attainment of satisfactory post-treatment outcomes may necessitate a protracted timeframe [8-10].

This study aimed to investigate the impact of social anxiety in conjunction with orthognathic surgery on the various components constituting OHRQoL.

## Materials and methods

In this study, secondary data were collated from prior cross-sectional and longitudinal research studies. Ethical approval was received from the Research Ethical Committee at the Faculty of Dentistry (REC-FDKAU), King Abdulaziz University, Jeddah, Saudi Arabia (protocol number: 182-11-19). The dataset incorporated information pertaining to 70 patients who were undergoing orthognathic treatment at the Dental School of King Abdulaziz University, Jeddah, Saudi Arabia, to facilitate an evaluation of their QoL before and after the surgical procedure. To accomplish this, participants were administered the Orthognathic Quality of Life questionnaire (OQLQ), an instrument initially formulated and validated by Cunningham et al. in 2002 to gauge the QoL of orthognathic surgery recipients [8,9]. Given that the study cohort consisted primarily of Arabic-speaking individuals, the OQLQ was translated into Arabic. Participants were also required to provide written informed consent, affording them a comprehensive understanding of the study's intricacies. Personal and clinical data encompassing factors like age, gender, history of orthodontic intervention, and prior orthognathic surgeries were documented. Participants were explicitly instructed to not include any identifying details, with a guarantee of the confidentiality of their responses.

The questionnaire was dispensed to patients across six distinct phases of treatment, namely: the pre-orthodontic phase, post-orthodontic phase, within 24 hours of the operation, 7-30 days post-operative, more than one month post surgery preceding the removal of braces, and the final visit post-brace removal. To facilitate data analysis, the six categories were amalgamated into three overarching phases: the pre-operative pre-orthodontic phase, the post-operative phase spanning 24 hours to 30 days, and the post-surgical phase spanning more than one month before and after the removal of braces.

The questionnaire encompassed seven chapters and featured a total of 42 questions, inclusive of 22 self-administered OQLQ items. Each chapter was thematically categorized. The first chapter centered on the patient's proficiency in executing normal oral functions such as mastication, respiration, and articulation. The second chapter honed in on the discomfort and pain experienced by the patient. The third chapter scrutinized the appearance and functionality of the teeth. The fourth chapter placed emphasis on the facial appearance of the patient. The fifth chapter delved into the psychological impact of the surgical intervention on the patient. The sixth chapter solicited insights into the patient's motivations for undergoing surgery, while the seventh and final chapter sought to ascertain patient satisfaction subsequent to treatment.

The questions were designed to evaluate two fundamental aspects: the frequency with which particular issues manifested and the extent to which these issues perturbed the patient. Responses were structured around a four-point scale and were subsequently regrouped for analytical purposes. Regarding frequency, the scale encompassed the following options: "sometimes," "most of the time," "always," and "never." In terms of the degree of bother experienced by the participant, response choices ranged from "does not bother me at all," "just a little," "sometimes," to "bothers me a lot." For the purposes of this study, responses classified as "bothers me a lot," "just a little," and "sometimes" were amalgamated into a singular category termed "to some degree," whereas "does not bother me" was allocated to a distinct category denoted as "not at all." Nine questions were extracted from the 22 self-administered OQLQ items, with a focus on social anxiety measured through the lens of "how much it bothers you." These nine questions were utilized as predictive variables, following which groups were stratified based on their quality-of-life components.

Subsequent to the analysis of these nine predictive variables, participants were partitioned into two distinct groups: those exhibiting social anxiety (to some degree) and those devoid of social anxiety (not at all). Additionally, scores derived from the four domains outlined in the OQLQ were evaluated and contrasted between these two groups. Data analysis was undertaken using PASW Statistics for Windows, Version 18.0 (Released 2009; SPSS Inc., Chicago, United States). Demographics and answers to the questions were tabulated as means and standard deviations. To assess the impact of the question component on participants' responses, we conducted a two-sample t-test. This analysis compared participants who reported being unaffected by the question component with those who indicated some level of concern. The t-test was performed for each of the four domains: social aspects of facial deformity (D1), facial aesthetics (D2), oral functions (D3), and awareness of dentofacial aesthetics (D4). These domains were examined for every item within the nine OQLQ social anxiety scale questions.

## Results

In this cross-sectional study, we examined the responses of 70 patients who willingly participated and completed the survey. Among them, the majority (n = 38, 54.29%) completed the survey more than one month after their surgery. Our participant demographic predominantly comprised females (n = 43, 61.43%), with ages ranging from 17 to 45 years. Detailed descriptive statistics and responses to the OQLQ social anxiety scale items are provided in Table 1.

**Table 1:** Responses to social anxiety scale items in the OQLQ and some demographic characteristics OQLQ: Orthognathic Quality of Life questionnaire

Variables	Frequency	Percent
Gender		
Male	27	38.57
Female	43	61.43
Stage of Treatment		
Pre-Surgery	30	42.86
Within 24 hours to 1 month	2	2.86
More than 1 month	38	54.29
Do you hate smiling (Because of the appearance of your teeth)?		
Not At All	21	53.85
To some degree	18	46.15
Have you ever felt concerned about not looking pretty like others?		
Not At All	21	55.26
To some degree	17	44.74
Do you hate being video recorded or photographed?		
Not At All	21	50
To some degree	21	50
Have you ever felt self-insecure?		
Not At All	23	71.88
To some degree	9	28.13
Are you concerned about what people think about you?		
Not At All	16	61.54
To some degree	10	38.46
Are you concerned about what people say about you?		
Not At All	22	64.71
To some degree	12	35.29
Are you concerned about having fewer chances in life? (Jobs, getting married)		
Not At All	14	66.67
To some degree	7	33.33
Are you concerned about meeting new people?		
Not At All	17	77.27
To some degree	5	22.73
Are you concerned about your health condition being less than others?		
Not At All	15	71.43
To some degree	6	28.57

The t-test results revealed multiple statistically significant differences between the two groups in several instances, including D1 in the question “Do you hate smiling (Because of the appearance of your teeth)?”, D1, D3, and D4 for the question “Have you ever felt concerned about not looking pretty like others?”, D2 in the question “Are you concerned about what people think about you?”, and D1 and D2 for the question “Are you concerned about what people say about you?.” These differences were statistically significant at P < 0.05. Detailed statistics, including means and standard deviations, can be found in Table 2.

**Table 2:** Comparison of participants who were not bothered by the queried component and participants who were bothered to some extent ^‡^: statistically significant at P < 0.05 D1: social aspects of facial deformity domain; D2: facial esthetics domain; D3: oral functions domain; D4: awareness of dentofacial esthetics domain

Variable	Not at all		To some degree	
	Mean	SD	Mean	SD
Do you hate smiling (Because of the appearance of your teeth)?				
D1: Social aspects of facial deformity^‡^	43.52	6.33	22.33	24.87
D2: Facial Esthetics	55.24	70.44	22.68	25.92
D3: Oral Functions	48.62	51.67	19.68	32.44
D4: Awareness of dentofacial esthetics	52.48	60.5	25.04	31.55
Have you ever felt concerned about not looking pretty like others?				
D1: Social aspects of facial deformity^‡^	41.95	67.76	19.5	25.14
D2: Facial Esthetics	55.25	69.65	27.62	22.63
D3: Oral Functions^‡^	37.85	58.41	17.62	33.82
D4: Awareness of dentofacial esthetics^‡^	45	68.35	27.24	27.52
Do you hate being video recorded or photographed?				
D1: Social aspects of facial deformity	44.81	54.35	22.52	25.71
D2: Facial Esthetics	56.38	64.7	25.6	23.91
D3: Oral Functions	44.33	51.65	19.08	32.03
D4: Awareness of dentofacial esthetics	50.67	58.75	24.02	30.26
Have you ever felt self-insecure?				
D1: Social aspects of facial deformity	51.23	67.33	27.14	17.11
D2: Facial Esthetics	62.82	77.56	27.32	16.06
D3: Oral Functions	41.23	65.67	18.74	41.32
D4: Awareness of dentofacial esthetics	53.05	67.22	28.79	30.01
Are you concerned about what people think about you?				
D1: Social aspects of facial deformity	50.63	64.9	21.83	20.96
D2: Facial Esthetics^‡^	53.25	77	21.99	20.25
D3: Oral Functions	45.88	57.3	19.81	41.31
D4: Awareness of dentofacial esthetics	49.06	64	27.76	29.89
Are you concerned about what people say about you?				
D1: Social aspects of facial deformity^‡^	48.45	73.83	21.34	19.84
D2: Facial Esthetics^‡^	57.18	75.5	25.72	17.6
D3: Oral Functions	45.41	58.25	21.23	37.4
D4: Awareness of dentofacial esthetics	50.55	70.17	26.94	28.37
Are you concerned about having fewer chances in life? (e.g., better jobs, getting married)				
D1: Social aspects of facial deformity	56.54	74.57	29.49	17.37
D2: Facial Esthetics	58.15	72.29	26.26	23.45
D3: Oral Functions	48.62	66	24.6	39.55
D4: Awareness of dentofacial esthetics	53.46	73.14	33.87	30.4
Are you concerned about meeting new people?				
D1: Social aspects of facial deformity	62.94	81.6	21.55	20.38
D2: Facial Esthetics	65.5	72.4	22.86	25.63
D3: Oral Functions	50.25	75.2	23.84	44.85
D4: Awareness of dentofacial esthetics	59.5	84	28.69	31.1
Are you concerned about your health condition being lower than others?				
D1: Social aspects of facial deformity	51.21	62.83	27.76	31.88
D2: Facial Esthetics	56.86	65	24.87	27.42
D3: Oral Functions	53.5	70	18.53	43.06
D4: Awareness of dentofacial esthetics	56.64	63.33	25.21	42.15

## Discussion

The primary objective of this study was to assess social anxiety across four domains (social aspects of facial deformity, facial aesthetics, oral functions, and awareness of dentofacial aesthetics) among two distinct participant groups categorized as socially anxious and non-anxious individuals. To our knowledge, no prior research has examined social anxiety and its association with the OHRQoL in these four specific domains. Furthermore, this study employed a sample population from Saudi Arabia, a region with limited existing literature on OHRQoL, social anxiety, and orthognathic surgery patients.

Our findings indicate variations in the impact of social anxiety on the four OQLQ domains. However, it's essential to note that some results did not achieve statistical significance. This might be attributed to the relatively small sample size of 70 participants or the presence of potential confounding variables. Additionally, patients' adaptation to their appearance over an extended period may influence our results without a discernible effect on their QoL measures.

A study by Sun et al. previously observed that patients with dentofacial deformities exhibited lower QoL compared to the healthy population, particularly in functional and psychological aspects [11]. Orthognathic surgery was shown to have a significant positive impact on their QoL, aligning with the outcomes observed in our study regarding the improvement in OQLQ domains between the two groups.

In a Saudi Arabian study conducted by Abdullah, the impact of orthognathic surgery on the QoL among Saudi patients was measured using a translated version of the OQLQ, incorporating all four components [12]. In our study, we expanded upon this by analyzing the social anxiety of orthognathic surgery patients in Saudi Arabia, assessed through nine selected questions, alongside the same four components of the OQLQ. Results reported by Abdullah in 2015 demonstrated similar findings regarding post-orthognathic surgery improvement in the QoL [12].

Additionally, a study by Hanafy et al. performed two types of orthognathic surgery and studied the QoL after both procedures [13]. Their conclusion highlighted that both groups exhibited significant enhancements in their QoL post-surgery, with no notable differences between the two techniques. Consequently, orthognathic surgery was found to enhance the QoL, regardless of the specific surgical approach.

In contemporary times, individuals are increasingly aware of the psychological aspects and their influence on daily routines and overall well-being [14]. They pay more attention to their facial and physical appearance, the quality of their interpersonal communication, and the subsequent effects on their overall QoL [15,16]. Our study aimed to quantify how these factors impact oral health and QoL, incorporating questions to assess the presence of social anxiety. However, it is crucial to acknowledge the limitations of our study, primarily stemming from the use of data from a previous database with a relatively small sample size. Therefore, larger sample sizes are recommended to yield more comprehensive and definitive results. Additionally, we propose exploring gender as a predictor of social anxiety and its potential confounding effect on OHRQoL. Further studies are warranted to investigate social anxiety, gender dynamics, orthognathic surgery outcomes, and other potential confounders on OHRQoL, employing robust longitudinal designs to minimize subject variability.

## Conclusions

The objective of this study was to assess the impact of patient social anxiety and orthognathic surgery on OHRQoL. Our findings indicate that both social anxiety and orthognathic surgery have a notable influence on patients' OHRQoL. We recommend further research to delve into the immediate and long-term effects of social anxiety and orthognathic surgery on the various subcomponents of quality of life. This deeper exploration will contribute to a more comprehensive understanding of these crucial factors and their implications for patients' well-being.
